# An E3 Ubiquitin Ligase Scaffolding Protein Is Proviral during Chikungunya Virus Infection in Aedes aegypti

**DOI:** 10.1128/spectrum.00595-22

**Published:** 2022-04-18

**Authors:** Sunil Kumar Dubey, Divya Mehta, Sakshi Chaudhary, Abdul Hasan, Sujatha Sunil

**Affiliations:** a Vector Borne Disease Group, International Centre for Genetic Engineering and Biotechnology (ICGEB), New Delhi, India; Wuhan Institute of Virology

**Keywords:** Chikungunya virus, *Aedes aegypti*, vector–virus interactions, ubiquitin proteasomal pathway

## Abstract

Chikungunya virus (CHIKV) is a reemerging alphavirus causing chikungunya disease (CHIKD) and is transmitted to humans by *Aedes* mosquitoes. The virus establishes an intricate balance of cellular interactions that ultimately helps in its replication and dodges cellular immune response. In an attempt to identify cellular host factors required during CHIKV replication in Aag2 cells, we performed global transcriptomics of CHIKV-infected Aag2 cells, and further, we compared this library with the *Drosophila* RNAi Screening Center (DRSC) database and identified transcripts that were regulated in Aedes aegypti during CHIKV infection. These analyses revealed specific pathways, such as ubiquitin-related pathways, proteolysis pathways, protein catabolic processes, protein modification, and cellular protein metabolic processes, involved during replication of the virus. Loss-of-function assays of selected candidates revealed their proviral or antiviral characteristics upon CHIKV infection in A. aegypti-derived Aag2 cells. Further validations identified that the ubiquitin proteasomal pathway is required for CHIKV infection in A. aegypti and that an important member of this family of proteins, namely, AeCullin-3 (*Aedes* ortholog of human cullin-3), is a proviral host factor of CHIKV replication in Aag2 cells.

**IMPORTANCE** Arboviruses cause several diseases in humans and livestock. Vector control is the main strategy for controlling diseases transmitted by mosquitoes. In this context, it becomes paramount to understand how the viruses replicate in the vector for designing better transmission blocking strategies. We obtained the global transcriptome signature of A. aegypti cells during CHIKV infection, and in order to obtain the maximum information from these data sets, we further utilized the well-characterized *Drosophila* system and arrived upon a set of transcripts and their pathways that affect A. aegypti cells during CHIKV infection. These analyses and further validations reveal that important pathways related to protein degradation are actively involved during CHIKV infection in A. aegypti and are mainly proviral. Targeting these molecules may provide novel approaches for blocking CHIKV replication in A. aegypti.

## INTRODUCTION

Chikungunya virus (CHIKV) is one of the reemerging zoonotic viruses endemic mainly to tropical and sub-Saharan regions. The virus is transmitted by mosquitoes belonging to the genus *Aedes*, with Aedes aegypti serving as the favored vector in the tropics and Aedes albopictus in temperate regions (1). Increased travel and trade have led to reemergence and large-scale epidemics in India, Africa, and Europe (1–3). Infection with CHIKV has been associated with extensive morbidity causing severe and self-limiting arthralgia in affected individuals that is sometimes chronic (4). Several approaches are under way for the development of an effective treatment against the infection. Despite the efforts, no effective therapeutics are developed that could limit pathogen spread and infectivity. Disease control strategies pertain mostly to demolishing vector breeding grounds and usage of insecticides (5, 6). Newer approaches involve developing genetically modified mosquitoes that could block viral transmission (7–9). Therefore, increased understanding of vector-virus interactome will help design newer and more effective strategies to restrict viral spread.

Host proteins play a significant role during viral replication that can be categorized as either proviral or antiviral depending on whether they facilitate or counter the establishment of virus in the host/vector. A number of elaborated studies in the last decade that were focused on CHIKV and host/vector interactions recognized a plethora of cellular processes and pathways that were of significance during infection (10–14). These included components of stress granules (15), mitochondria (13), and endoplasmic reticulum (16). Thus, by virtue of modulating these essential cellular proteins, the virus becomes capable of hijacking cellular responses enabling its efficient replication.

High-throughput screening and computational analyses are some of the tactics that are routinely employed to identify cellular factors. These studies provide a global perspective for the role of the host genes in viral infection. The present study is one such study where we identified A. aegypti host factors involved in CHIKV replication. We first performed global transcriptomic analysis of an A. aegypti cell line post CHIKV infection and identified transcripts that were regulated during CHIKV replication. In addition, we selected known genes from the *Drosophila* RNAi Screening Center (DRSC) database and performed a hybrid analysis to identify transcripts that may be involved in arboviral replication in the vector. The identified transcripts were evaluated for their role in CHIKV replication through a double-stranded RNA (dsRNA)-mediated RNAi screen. Finally, A. aegypti ortholog of human cullin-3 protein, referred to here as AeCullin-3, was found to be proviral during CHIKV replication.

## RESULTS

### Identification of Aedes aegypti host factors involved during infection with CHIKV.

**FIG 1:**
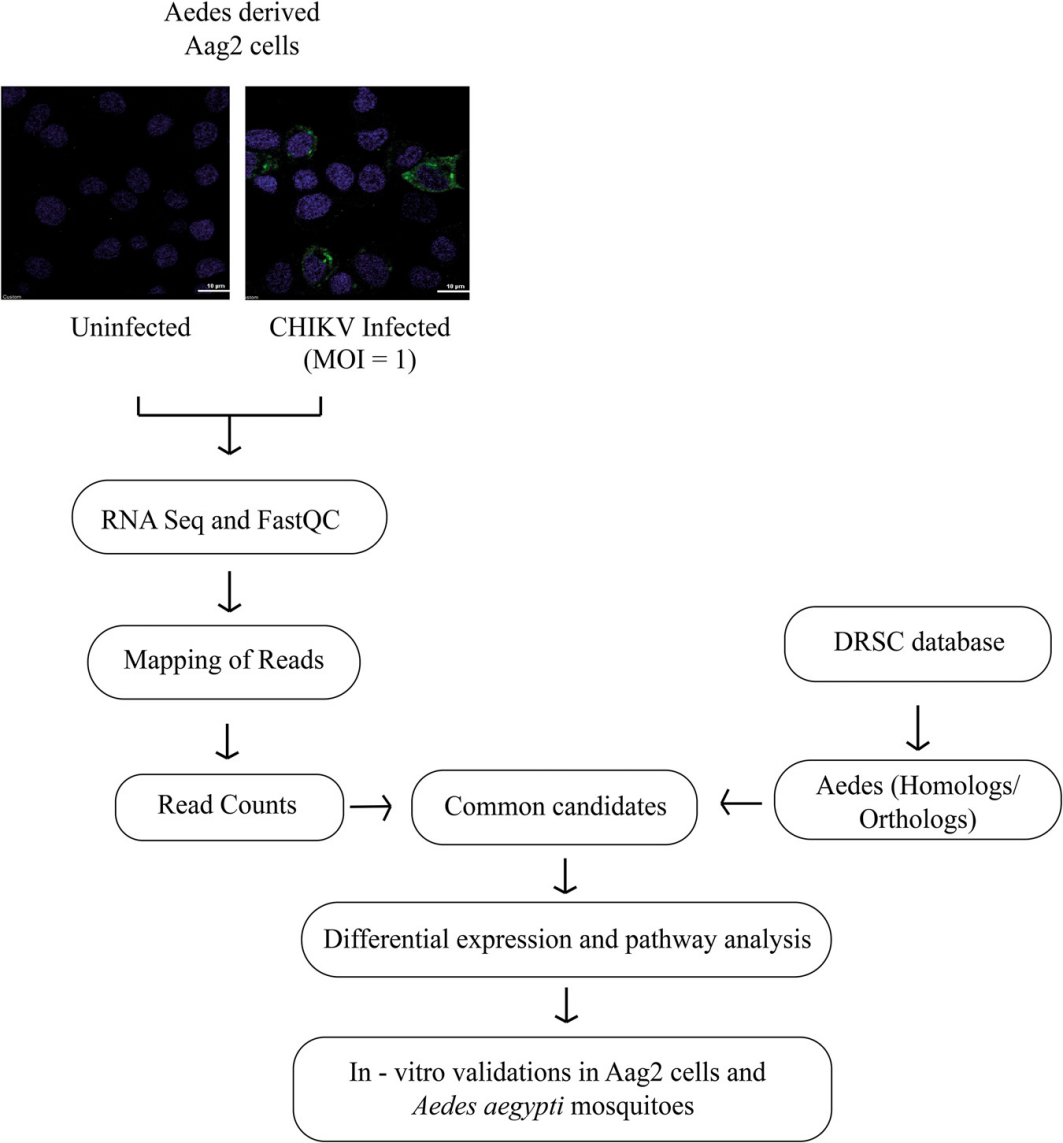
Pipeline used for the identification of Aedes aegypti host factors during infection with CHIKV.

The pipeline used for the identification of A. aegypti host factors is depicted in Fig. 1.

**FIG 2:**
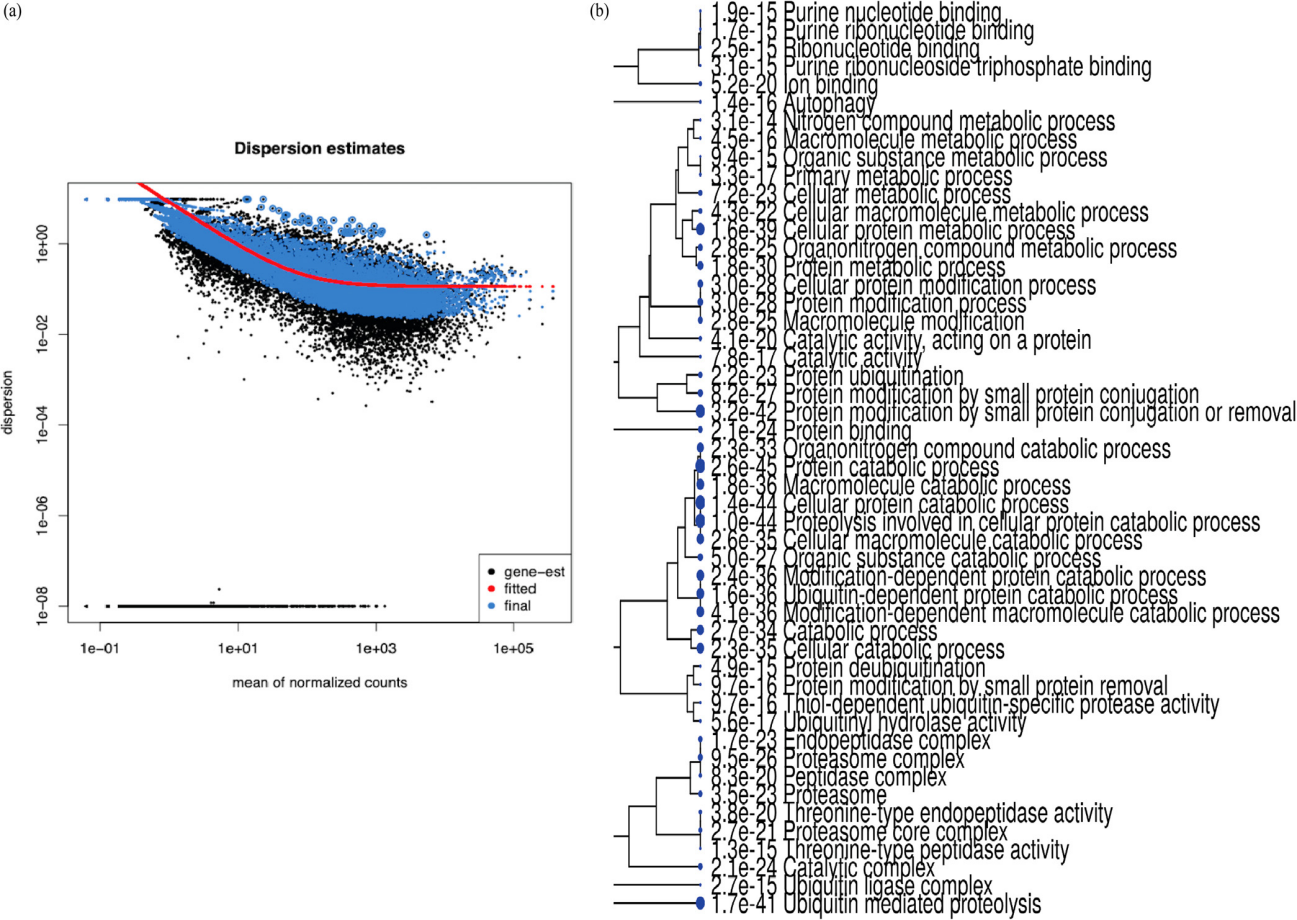

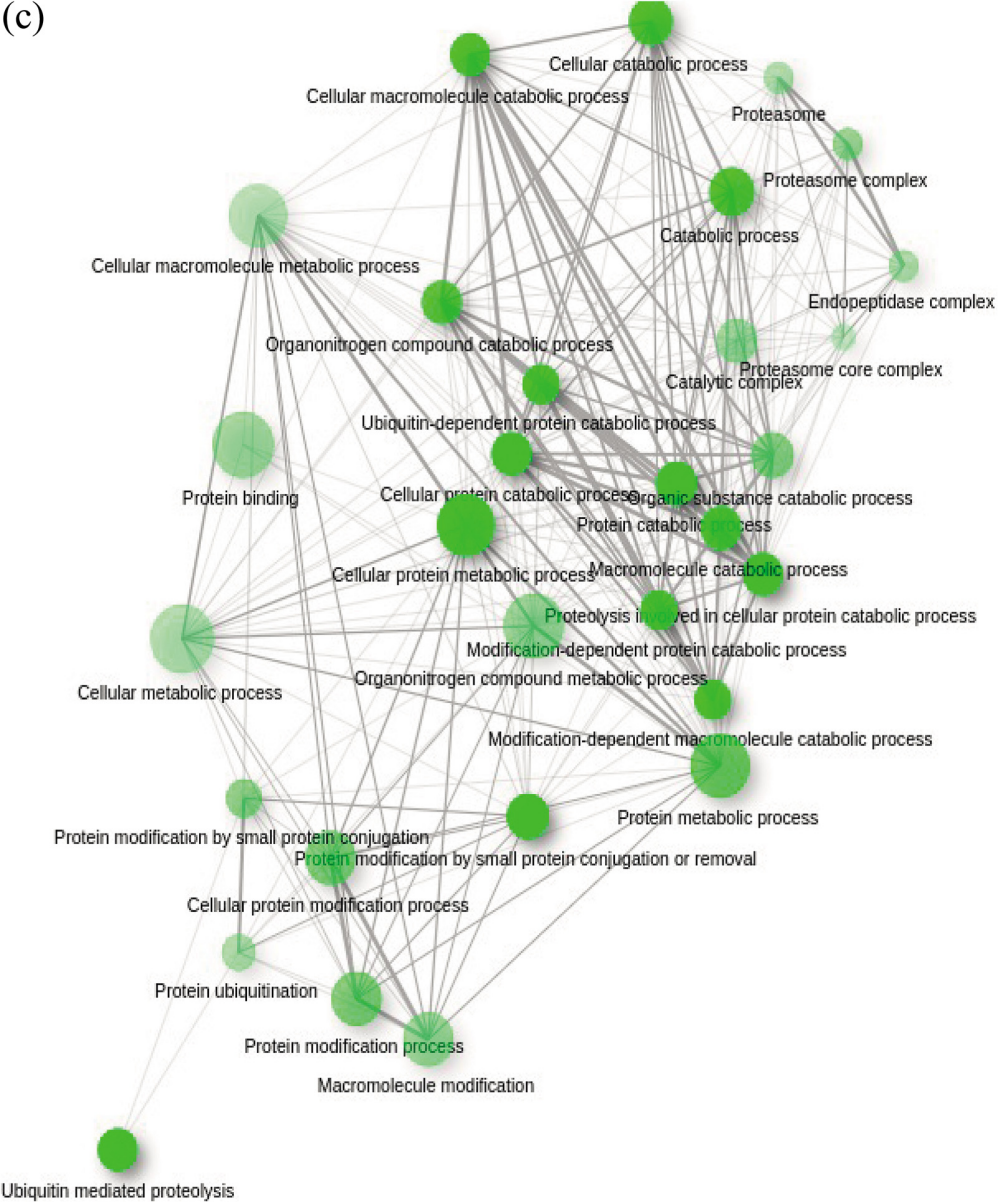
Identification of putative A. aegypti host factors upon CHIKV infection. (a) Representation of differentially expressed genes between two libraries. Black dots represent overall differentially expressed genes while blue dots represent significantly expressed genes (Fold change ± 1.5 and *P* value < 0.05). (b) Pathway analysis of all 8,608 transcripts found common between lab-generated transcriptomics libraries and DRSC database. Transcripts from pathways such as ubiquitin-related pathways, proteolysis pathways, protein catabolic processes, protein modification, and cellular protein metabolic processes were significantly affected upon CHIKV infection of Aag2 cell line. The sizes of the blue dots represent significance, with bigger dots having a lower *P* value. (c) Functional analysis of identified orthologous 346 genes found from significant selected pathways. Darker nodes are more significantly enriched gene sets. Bigger nodes represent larger gene sets. Thicker edges represent more overlapped genes.

RNA-Seq data acquired by processing of the uninfected and infected libraries (done in triplicates) were processed for quality trimming and removal of adapter sequences before proceeding with mapping to A. aegypti genome. The mapping percentage for all samples was found to be above 80%, and the total number of transcripts identified in each individual library varied between 12,000 to 15,000 (Table 1, Fig. 2a).

**TABLE 1:** RNA-Seq data processing: reads obtained after trimming and mapping percentage and number of identified transcripts

Sample name	No. of reads after trimming (bp)	Mapping percentage (%)	No. of identified transcripts
Aag2_infected_1	32,897,025	84.39%	14,179
Aag2_uninfected_1	67,948,278	84.15%	14,893
Aag2_infected_2	20,229,369	85.33%	12,716
Aag2_uninfected_2	24,229,683	84.10%	12,987
Aag2_uninfected_3	22,723,606	87.73%	12,687
Aag2_infected_3	20,948,346	85.31%	12,704

To further explore the vector-virus interactome within the host cell, we utilized the extensive information available on the model insect, namely, *Drosophila*, to better annotate the *Aedes* genes. From the early 1900s to the present, Drosophila melanogaster has been central to groundbreaking research in diverse organisms ranging from humans to insects (17–19). Previous studies have performed hybrid high-throughput screens of *Drosophila* genes, including RNAi screens, to understand host-pathogen interactions in their organisms of interest and have established different protocols to perform the same (17, 20). In the present study, using a similar approach, we compared our transcriptome libraries with *Drosophila* RNAi Screening Center (DRSC) database. Comparison of the *Drosophila* data sets with our transcriptome data presented a total of 8,608 transcripts that were found to be common and were taken for further analysis. Pathway analysis revealed that ubiquitin-related pathways, proteolysis pathways, protein catabolic processes, protein modification, and cellular protein metabolic processes were significantly affected (Fig. 2b and c). Differential expression analysis revealed 346 genes to be differentially expressed, of which 34 genes were found to be significant (*P* value of <0.05 and/or fold change difference of ±1.5) (Data set S1). This set of genes was further reviewed for genes’ pathways, and 7 genes were taken for downstream validation.

### dsRNA-based screening to elucidate the proviral or antiviral role of identified cellular factors.

In addition to the above 7 genes, we performed a literature search for putative host factors in insects that played a role in infection and cellular physiology and selected 23 additional genes for a dsRNA-based screening to evaluate their role in CHIKV infection in A. aegypti cells (Table 2). The final set of 30 transcripts for dsRNA screening were involved in cellular processes, including metabolism, biosynthesis, and oxidative pathways (AAEL001895 [21], AAEL013361 [22, 23], AAEL013603 [24], AAEL003977, AAEL014840 [25], AAEL001292 [26], AAEL012868 [27]), protein modification and proteolysis (AAEL011287, AAEL007187, AAEL019450, AAEL006797 [28], AAEL017567 [29], AAEL003104 [30], AAEL010641, AAEL001112, AAEL012337), transcriptional/translational regulatory and nucleic acid binding proteins (AAEL019736 [31], AAEL019431, AAEL008073, AAEL007945, AAEL001612), and ion channels, receptors, cell adhesion proteins, and transmembrane proteins (AAEL002922, AAEL009229, AAEL012421, AAEL005681 [32], AAEL005133, AAEL008641, AAEL006685). One transcript was found to be involved in endocytosis (AAEL007041 [32]). Apart from these, one uncharacterized transcript was also included in the study (AAEL024345).

**TABLE 2:** Detailed information on the putative host factors selected for dsRNA-based screening; the description shown is acquired from Uniprot (83) and Vectorbase (72)

Gene ID	Gene name	Gene ontology
AAEL011287	Ubiquitin specific protease 1	Protein deubiquitination
AAEL014840	Short change dehydrogenase	Oxidoreductase activity
AAEL001895	Alpha-1,4-galactosyltransferase	Glycan biosynthesis and metabolism
AAEL001112	Ubiquitin carboxyl-terminal hydrolase	Protein deubiquitination
AAEL019736	HTH CENPB-type domain-containing protein	DNA binding
AAEL007187	Cullin ubiquitin ligase	E3 ubiquitin ligase
AAEL019431	Ecdysone receptor	DNA-binding transcription factor activity
AAEL002922	Ionotropic receptor 8a	Ion channel
AAEL019450	Peptidase_S8 domain-containing protein	Protease
AAEL008641	GTP-binding protein (o) alpha subunit, gnao	G protein coupled receptor
AAEL008073	Putative mRNA binding protein	RNA binding
AAEL013603	Short-chain dehydrogenase	Oxidoreductase activity
AAEL013361	Lipase	Hydrolase activity, lipid catabolic process
AAEL009229	Mitochondrial citrate transport protein, putative	Transmembrane transport
AAEL003977	Very-long-chain 3-oxoacyl-CoA synthase	Fatty acid biosynthesis
AAEL012421	Cadherin	Cell-cell adhesions
AAEL010641	SUMO-activating enzyme subunit	Protein sumoylation
AAEL006797	F-box and leucine-rich repeat protein 7	Ubiquitination
AAEL005681	GPRHIS	G protein coupled receptor
AAEL007945	Eukaryotic translation initiation factor 3 subunit H	Translational initiation factor activity
AAEL001612	Dicer-1	Posttranscriptional gene silencing by RNA
AAEL017567	Metalloprotease	Hydrolase/metallopeptidase activity, proteolysis
AAEL024345	Uncharacterized protein	Uncharacterized protein
AAEL012337	Goliath E3 ubiquitin ligase	E3 ubiquitin ligase
AAEL012868	Cmp-n-acetylneuraminic acid synthase	Metabolism
AAEL001292	Cytochrome P450	Oxidoreductase activity
AAEL003104	Tripartite motif protein trim 2,3	Metal ion binding, ubiquitination
AAEL005133	Tetratricopeptide repeat protein, tpr	Nonmotile cilium assembly
AAEL007041	Low-density lipoprotein receptor (ldl)	Calcium ion binding, endocytosis
AAEL006685	Guanine nucleotide-binding protein subunit gamma	G protein coupled receptor

**FIG 3:**
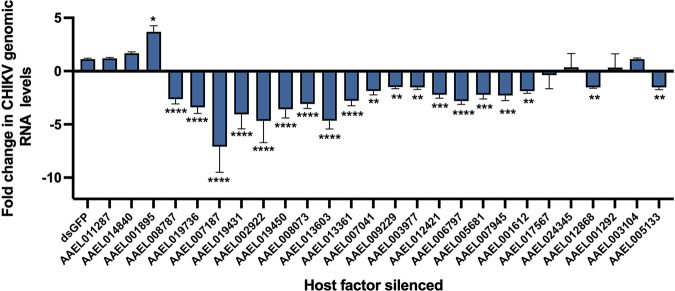
RNAi screening for A. aegypti host factors in CHIKV replication. Quantitative real-time PCR (qRT-PCR) analysis of CHIKV genomic RNA levels upon dsRNA-mediated silencing followed by CHIKV infection. Rps17 was used as an endogenous control and data are analyzed using 2^−ΔΔ^*^CT^*. Data are expressed as mean ± SD; ****, *P < *0.0001 versus control group; ***, *P* < 0.001 versus control group; **, *P* < 0.01 versus control; *, *P* < 0.1 versus control.

The selected transcripts were silenced using dsRNA after estimating their silencing efficiencies (14). dsRNAs showing a silencing efficiency of >50% were taken further for the loss-of-function studies (data not shown). A known host factor, V-type proton ATPase catalytic subunit A, that was previously reported to be involved in arboviral infections was also taken and served as the positive control (AAEL008787) (33), and dsRNA against GFP served as the negative control. Five genes (AAEL012337, AAEL006685, AAEL008641, AAEL010641, AAEL001112) could not be optimized for silencing and were therefore not included further. Screening of the selected transcripts during CHIKV infection using dsRNA-mediated silencing in A. aegypti-derived Aag2 cells revealed that CHIKV viral RNA levels differed significantly among cells with silenced transcripts compared to those in the negative control (GFP dsRNA-transfected cells) upon CHIKV infection. The relative CHIKV genomic RNA level for AAEL011287, AAEL014840, and AAEL001895 were found to be higher than that of the negative control. Among these factors, AAEL001895 (4-alpha-galactosyltransferase) was the most significant one exhibiting antiviral activity. Viral genomic RNA levels showed no significant change for six genes relative to negative control, namely, AAEL011287, AAEL014840, AAEL017567, AAEL024345, AAEL001292, and AAEL003104. Genes AAEL019736, AAEL007187, AAEL019431, AAEL002922, AAEL019450, AAEL008073, AAEL013603, AAEL013361, AAEL007041, AAEL009229, AAEL003977, AAEL012421, AAEL006797, AAEL005681, AAEL007945, AAEL001612, AAEL012868, and AAEL005133 all showed reduction in relative CHIKV genomic RNA level, suggesting potential proviral activity. Among these factors, AAEL007187 (AeCullin-3) was the most significant factor showing the proviral activity for CHIKV (Fig. 3).

### Proteasomal pathway is required for CHIKV replication in Aag2 cells post entry.

Previous studies have established a functional role for ubiquitin proteasomal machinery during viral replication in host cells (as reviewed in references 34 and 35). Studies done on alphaviruses have employed proteasome inhibitors to decipher the function of ubiquitin proteosome pathway (UPP) machinery (36–38). Global proteomic analysis of CHIKV-infected host cells has also revealed several components of the proteasome to be differentially regulated (39). In our initial analysis, we found the protein modification pathway to be evidently involved during the process of CHIKV replication. Transcripts for proteins such as proteases, ubiquitin ligases, and deubiquitinases were found to be significantly regulated in Aag2 cells upon infection. E3 ubiquitin ligase belonging to the TRIM family (AAEL003104) showed no significant impact on CHIKV replication upon being silenced. The F-box and leucine-rich repeat protein 7 (which functions as an adaptor for SCF E3 ligase; AAEL006797) along with cullin-3 (now referred to as AeCullin-3) were found to be proviral for CHIKV replication.

**FIG 4:**
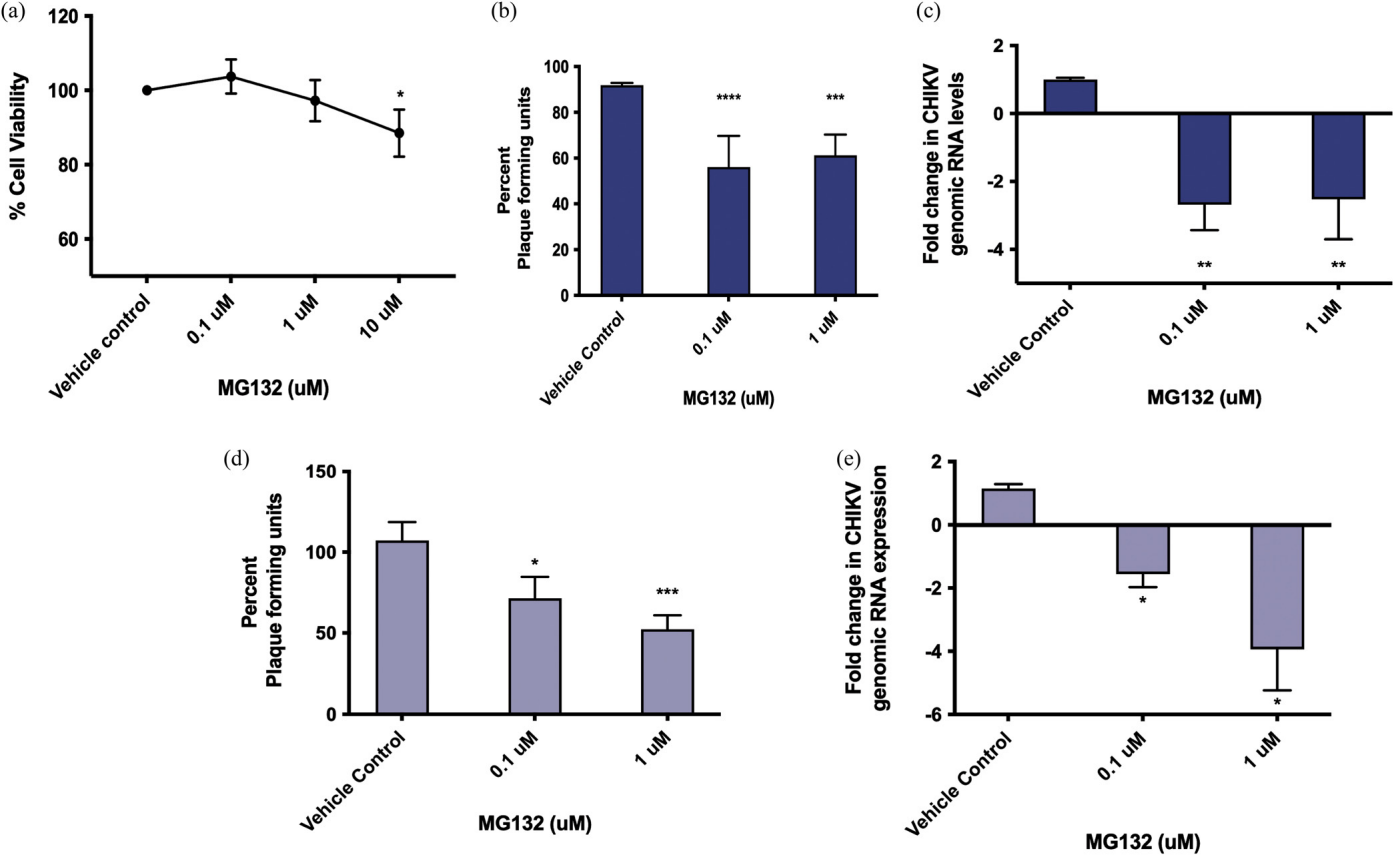
AeCullin-3 is proviral during CHIKV replication in Aag2 cells. (a) MTT assay to determine percentage of viable cells in the presence of MG132 at 0.1 μM, 1 μM, and 10 μM. One percent DMSO was used as vehicle control. (b) Plaque assay for viral titer determination in MG132 - CHIKV pretreatment assay. (c) qRT-PCR for CHIKV genomic RNA levels in MG132-CHIKV pretreatment assay. (d) Plaque assay for viral titer determination in MG132-CHIKV post-treatment assay. (e) qRT-PCR for CHIKV genomic RNA levels in MG132-CHIKV posttreatment assay. Rps17 was used as an endogenous control in qRT-PCR, and the results were analyzed using 2^−ΔΔ^*^CT^*. The experiments were performed a minimum of three times, with each experiment set in triplicates. Data are expressed as mean ± SD. ****, *P* value < 0.0001; ***, *P* < 0.001; **, *P* < 0.01; *, *P* < 0.1 (versus control).

To elucidate if there was any role of proteasomal pathway in CHIKV replication, we used a well-known proteasome inhibitor MG132 in pre and posttreatment assays. MG132 has also previously been employed as a proteasome inhibitor in Aag2 cells (40, 41). Prior to the assay, cell viability upon MG132 treatment was tested in Aag2 cells, and it was found that the cell viability remained above 80% at the three drug concentrations tested, namely, 0.1 μM, 1 μM, and 10 μM (Fig. 4a). Based on the results, 0.1 μM and 1 μM were used for the inhibitor assays. Pretreatment assay determined whether proteasomal pathway had a significant involvement during early stages of viral entry and internalization. A significant reduction of approximately 40% (*P* < 0.0001) was observed in PFU at both 0.1 μM and 1 μM concentrations of MG132 (Fig. 4b). These results were also reproduced using quantitative real-time-PCR (qRT-PCR), and a 2-fold reduction (*P* < 0.01) was observed at both these concentrations (Fig. 4c). A posttreatment assay, on the other hand, determined the possible involvement at a postentry stage of viral life cycle within the cells. It was observed that during posttreatment of the drug, viral inhibition was dose dependent. While at 0.1 μM concentration a 30% reduction was obtained in plaque forming units (PFU/μL; *P* < 0.05), we observed a 50% reduction in viral genomic RNA levels at 1 μM concentration of MG132 (*P* < 0.001) (Fig. 4d). Similar results were also obtained in qRT-PCR, showing a 2-fold reduction at 0.1 μM (*P* < 0.05) and 4-fold reduction at 1 μM concentration of MG132 (*P* value < 0.05) (Fig. 4e).

### Role of AeCullin-3 during CHIKV infection in A. aegypti.

**FIG 5:**
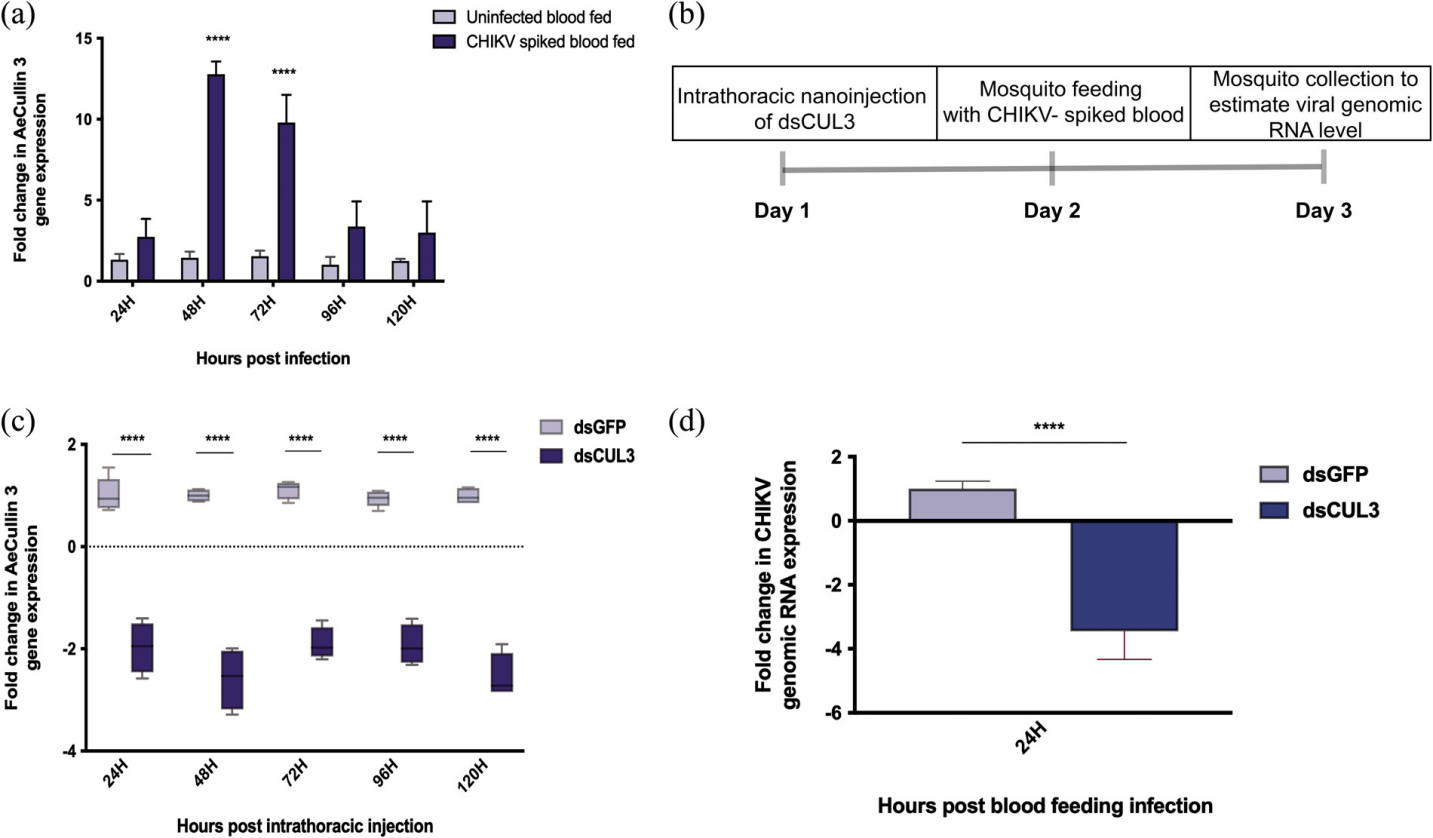
Role of AeCullin-3 during CHIKV infection in A. aegypti. (a) A. aegypti mosquitoes were fed with either uninfected blood (control group) or CHIKV-spiked blood (10^6^ PFU/mL), a pool of *n* = 5 mosquitoes was collected at indicated time points in TRIzol for RNA extractions. AeCullin-3 gene expression was quantified in the two groups by qRT-PCR. The experiments were performed a minimum of three times, with each experiment set in triplicates. Fold change in expression in CHIKV-spiked blood-fed mosquitoes is compared to that in uninfected blood-fed mosquitoes. (b) Experimental plan for dsRNA-mediated knockdown of AeCullin-3 in A. aegypti mosquitoes. (c) Mosquitoes were intrathoracically injected with either dsRNA against GFP (dsGFP, control group) or dsRNA against AeCullin-3 (dsCUL3 group), and the mosquitoes (*n* = 5) from both groups were then collected and AeCullin-3 gene expression was estimated in each individual mosquito using qRT-PCR. (d) dsGFP- and dsCUL3-injected mosquitoes were subjected to CHIKV-spiked blood feeding 24 h post nanoinjection. Mosquitoes (*n* = 5) were then collected and CHIKV genomic RNA levels were analyzed in each individual mosquito. (a to d) Rps17 served as endogenous control, and qRT-PCR analysis for experiments indicated in this figure was done using 2^−Δ^*^CT^* method. Fold change in expression in dsCUL3-injected mosquitoes is compared to that in dsGFP-injected mosquitoes. Data are expressed as mean ± SD. ***, *P* < 0.001; **, *P* < 0.01; *, *P* < 0.1 (versus control).

In order to further validate the relevance of AeCullin-3 in CHIKV infection, we performed a dsRNA-based silencing in A. aegypti mosquitoes. First, expression levels of AeCullin-3 were determined using qRT-PCR in normal blood-fed and CHIKV-spiked blood-fed female A. aegypti individuals. AeCullin-3 expression levels increased to up to 10-fold at 48 h and starting at 72 h postinfection gradually returned to normalcy (Fig. 5a).

We then went on to further characterize the role of AeCullin-3 by performing a loss-of-function assay on the mosquitoes (Fig. 5b). Four- to six-day-old female A. aegypti individuals were injected with dsRNA against green fluorescent protein (GFP; control) or AeCullin-3. As seen in Fig. 5c, a 2-fold reduction in AeCullin-3 expression levels was obtained in all the time points tested (from 24 h to 120 h post nanoinjection). Based on this, 24-h post nanoinjection of both GFP and AeCullin-3, the mosquitoes were subjected to CHIKV infectious blood meal (10^6^ PFU/mL). In dsAeCullin-3-injected mosquitoes, a reduction of more than 3-fold in CHIKV genomic RNA levels (*P* < 0.0001) was obtained compared to those in the GFP control group (Fig. 5d), thereby confirming that AeCullin-3 acts as a proviral CHIKV host factor in A. aegypti mosquitoes.

## DISCUSSION

While much research has been executed to identify cellular factors of the mammalian host that play important roles in CHIKV replication (42–45), limited literature is available with respect to host factors regulated upon CHIKV infection in *Aedes* (13, 46–48). A deficit *Aedes*-specific database for genes/proteins is one of the major reasons for the dearth of in-depth research in this area. Despite the lack of a proper repertoire, RNA-Seq-based studies on A. aegypti (49–51) as well as A. albopictus (52) have often been employed to study vector-virus infection. These studies have helped in deciphering transcript patterns in response to monoinfections and coinfections (51) in midgut upon infection (49) and also in salivary glands (50).

Researchers have also resorted to computational analyses and better studied insect systems such as *Drosophila* to identify orthologs and *Aedes* factors that could affect alphavirus infection (19). Combining the knowledge available on the *Drosophila* system with lab-generated A. aegypti transcriptome data, and with further validations, our study unravels the role of the protein modification pathways such as ubiquitin-proteasome pathway (UPP) during CHIKV infection in Aag2 cells. Other than UPP, protein catabolic processes as well as cellular protein metabolism were found to be significantly affected during CHIKV infection, as has been emphasized by previous reports (11, 36, 39). In our analysis, we found that a major proportion of genes were part of the UPP, the pathway that regulates cellular protein turnover by an intricate interplay of three diverse enzymes, E1 ubiquitin activating enzyme, E2 ubiquitin conjugating enzyme, and E3 ubiquitin ligase. Briefly, the E1 enzyme activates ubiquitin by ATP to a high-energy intermediate followed by transfer of ubiquitin from E1 to substrate which is mediated by the E2 and E3 enzymes. Cellular proteins with covalently attached ubiquitin are targeted for degradation in the 26S proteasome (53).

Ubiquitin proteasomal pathway is considered to be one of the most important regulatory machineries in the cellular system. The machinery governs biological processes such as cell cycle regulation, apoptosis, transcription, translation, and cell signaling and therefore serves as an attractive target for viral manipulation (54). Viruses are often found to subvert ubiquitin proteasomal machinery by redirecting the E3 ubiquitin ligases to degrade host proteins in order to ensure survival and promote viral replication and dissemination (as reviewed in references 55 and 56). Using both proteasomal inhibitors and performing loss-of-function studies, our present study corroborates with these earlier studies and identified AeCullin-3 to be an important proviral host factor during CHIKV infection in both Aag2 cells and A. aegypti mosquitoes.

AeCullin-3 is known to serve as a scaffolding protein for the cullin-RING E3 ubiquitin ligase (CRUL). CRULs are the largest family of E3 ubiquitin ligases and are distinguished by the presence of a structural motif called RING domain that forms the interface for recruiting E2-conjugating enzymes (57). There are eight mammalian homologues for cullin, each pertaining to different substrate specificities. Viral proteins are often found to interact with CRULs (cullin-RING ubiquitin ligases) to subvert the machinery to newer cellular targets or to protect existing targets thus modulating protein turnover (55). Elaborate studies in the past two decades have established a number of downstream effects of CRULs subversion that include (i) degradation of proteins of host defense mechanism, (ii) degradation of cellular proteins to promote oncogenic transformation, and (iii) degradation of cell cycle regulatory proteins.

Viruses are known to encode proteins that interact with CRULs, as seen in Ectromelia virus and Vaccinia virus, where they encode proteins that contain domains structurally similar to the one present in cullin-3 adapters, and by virtue of the domain (BTB Kelch), viral proteins are able to interact with this sophisticated machinery and bring about changes in cellular physiology (58, 59). Similarly, viral proteins are known to participate in the subversions as reported in paramyxoviruses (60–63). All these studies, irrespective of the cullin homolog involved in the process, seem to target the STAT proteins. STAT dimerization is an important messenger for downstream activation of interferon (IFN) response, which might explain the subversion of different cullin homologues during virus replication. In the case of arboviruses, a study suggests that West Nile virus, when present in *Culex* mosquitoes, also adopts this subversion mechanism of CRULs for STAT degradation (64). However, unlike in the case of flaviviruses, immunity during an alphavirus infection in A. aegypti is imparted mainly by RNAi defenses, and other pathways such as JAK STAT and Toll/Imd have limited roles to play (65). Based on these findings, it would be interesting to identify whether subversion of A. aegypti cullin (AeCullin 3)-based E3 ubiquitin ligases is responsible for the nominal involvement of JAK STAT pathway during alphavirus infections. Apart from STAT proteins, viruses also divert CRULs to target other proteins of host defense, as well, such as in the case of HBV, where HBx protein mediates the degradation of antiviral protein through cullin-4-based CRULs (66). During retroviral infection, as well, HIV vif protein hijacks cullin 1 machinery to degrade APOBEC3G and promote viral replication (67). Oncogenic viruses also utilize cullin homologues to induce transformations; a well-known example includes SV40 LT antigen, which inhibits cullin-7 and increases downstream signaling pathway, ultimately leading to tumorigenic transformation (68). Thus, based on their requirements, viruses of different families often interact with CRULs in order to facilitate the degradation of host defense proteins or, in the case of retroviruses, promote oncogenic transformation. In the present study, we observed that within the A. aegypti-derived Aag2 cells, presence of AeCullin-3 promoted viral replication. We do hypothesize that CHIKV might be subverting CRUL-3 for degradation of host defense proteins; however, further studies are required to decipher the exact mechanism of action.

### Conclusion.

In summary, based on integrative analysis using global transcriptomics of A. aegypti-derived Aag2 cells upon CHIKV infection and *Drosophila* database screening, AeCullin-3 was identified and validated as one of the major and important host factors differentially regulated upon CHIKV infection in A. aegypti. Our study also revealed that the novel host factor has a proviral role during the postentry stage of viral replication.

## MATERIALS AND METHODS

### Cell culture and virus maintenance.

A. aegypti cell line (Aag2) was a kind gift from Alain Kohl (University of Glasgow, Scotland, UK). The cells were maintained at 28°C in Leibovitz medium (L-15; cat no. AL011S, HiMedia) supplemented with glutamine, tryptose phosphate broth, penicillin/streptomycin, and 10% fetal bovine serum (FBS). Vero cells (ATCC-CCL-81) and C6/36 cells were used for chikungunya virus propagation and were maintained in Dulbecco’s modified Eagle medium (DMEM; cat no. AL007A, HiMedia) supplemented with 10% FBS, penicillin/streptomycin, and glutamine at 37°C and 5% CO_2_.

Chikungunya virus (accession no. JF950631.1) was isolated from patient sample collected during the 2010 outbreak in India (69) and was propagated in alternate cycles in C6/36 cells and Vero cells. Virus propagated in Vero cells was collected after 48 h postinfection for determination of titer and subsequently used for infection in Aag2 cells.

### Sample preparation and RNA sequencing.

Briefly, Aag2 cells were seeded in two 6-well plates. Cells from one plate were incubated with CHIKV at a multiplicity of infection (MOI) of 1, and cells from the other were used as uninfected control. Twenty-four hours postinfection, cells were collected in TRIzol reagent (Invitrogen). RNA isolation was subsequently performed using TRIzol method (70). For RNA-Seq, three samples from uninfected control cells and three samples from CHIKV-infected cells were used. RNA-Seq was performed on total RNA using Illumina HiSeq 4000 at NGB Diagnostics Pvt. Ltd. (Noida, UP, India).

### Transcriptome assembly and read mapping.

The sequences from the reads of all the libraries were trimmed using Cutadapt (71) and FASTX toolkit (version 0.013) (http://hannonlab.cshl.edu/fastx_toolkit). Further quality check was performed on the trimmed sequences using FastQC tool (https://www.bioinformatics.babraham.ac.uk/index.html). The high-quality reads with a quality score of ≥20 were retained for further analysis. High-quality reads were mapped on A. aegypti genome (AaegL5.3) downloaded from VectorBase (https://vectorbase.org/vectorbase/app) (72) using STAR tool (73) to align the RNA-Seq reads. featureCounts (74) and the DESEQ2 package (75) were used for further analyses like count identification and differential expression analysis studies. The annotations of identified transcripts were also fetched from the VectorBase database using BioMart tool.

### Identification of putative host factors from analysis of public databases.

The RNA-Seq data were supplemented with publicly available data from the *Drosophila* DRSC (76) to identify well-annotated host factors of A. aegypti upon CHIKV infection. (Fig. 1b). The gene/transcript IDs of *Drosophila* were converted into A. aegypti ID using g:Profiler toolkit https://biit.cs.ut.ee/gprofiler/gost (77). Host factors from data mining and differential expression of Aag2 cells from RNA-Seq analyses were compiled and common significant transcripts were selected for gene ontology analysis, performed using g:profiler, ShinyGO (v0.741) (78), and webgestalt 2019 (http://www.webgestalt.org) (79). The databases and the toolkits were accessed during January 2021 to November 2021. Further, based on the functional annotation and characterization, putative host factors for CHIKV replication were selected for dsRNA screening.

### Double-stranded RNA preparation and transfection.

For *in vitro* transcription (IVT), T7 and SP6 polymerase were used for dsRNA preparation. pGEMT-easy vector was used for cloning genes for generating dsRNA. After IVT, the dsRNAs were purified using the TRIzol method. Purified dsRNAs were then used for transfections in Aag2 cell lines using transfection reagent Attractene (Qiagen). For transfections, 2 × 10^4^ to 8 × 10^4^ cells were seeded into 24-well plates in replicates. In each well, 400 ng of each dsRNA was transfected when 70% confluence was reached, along with negative control. After 4 h of incubation, the transfection medium was changed and fresh medium was added to the wells. Post 24 h, cells from one set of plates were collected to check silencing of the factors. The other plates were infected with CHIKV at an MOI of 1 and further processed for primary and secondary screening. dsRNAs were designed using snapdragon (80) primer details in Data set S2.

### RNAi screening of putative host factors using qRT-PCR.

Silencing efficiencies of dsRNA were estimated before setting up the experiments by transfecting cells with dsRNA and analyzing gene expression of the target transcript using qRT-PCR. Cells were transfected with 400 ng dsRNA 24 h prior to CHIKV infection and collected at 24 h postinfection. Further, RNA was isolated using TRIzol reagent (Invitrogen) per manufacturer’s instructions and was used for qRT-PCR analysis using QuantiTect SYBR green PCR kit (Qiagen, Germany). To estimate CHIKV genomic RNA, E1 gene was used and Rps17 served as endogenous control. Experiments were conducted a minimum of three times, with each experiment set up in triplicates. Expression levels were calculated using the 2^−ΔΔCT^ method (81).

### Cell viability using MTT assay.

Aag2 cells were seeded in a 96-well format at a density of 20,000 cells per well and incubated at 28°C overnight. MG132 (Sigma; dissolved in dimethyl sulfoxide [DMSO]) was used at 0.1, 1, and 10 μM (final concentration of DMSO, 1%) in serum-free L-15 medium and incubated at 28°C for 24 h. Cells were washed with phosphate-buffered saline (PBS), and 3-(4,5-dimethyl-2-thiazolyl)-2,5-diphenyl-2H-tetrazolium bromide (MTT) reagent (0.5 mg/mL) was added to each well, after which the cells were incubated for 4 h at 37°C. Cells were again washed, 100 μL DMSO was added to each well to dissolve MTT crystals, and cells were incubated at 37°C for 30 min. Absorbance was recorded at 570 nm and was used to calculate percent cell viability.

### Pretreatment and posttreatment assays with proteasomal inhibitor.

MG132 (Sigma) proteasome inhibitor was used for suppression of proteasomal machinery. For pretreatment assay, cells were initially treated with 0.1 μM and 1 μM drug concentrations with 1% DMSO as a vehicle control and incubated at 28°C for 24 h in 2% FBS-containing L-15 medium, which was followed by washing and CHIKV infection (MOI = 1) for 2 h. Cells and supernatants were collected at 24 h for qRT-PCR (viral genome quantification using primers for CHIKV E1 gene) and plaque assay (determination of viral titer), respectively.

For posttreatment assay, cells were infected with CHIKV (MOI = 1) for 2 h, washed, and then treated with MG132 at 0.1 μM and 1 μM concentrations again using 1% DMSO as vehicle. After a 24-h incubation, cells and supernatant were collected for qRT-PCR and plaque assays, respectively.

### Plaque assay.

Vero cells were plated in a 96-well format in DMEM containing 10% FBS and were incubated overnight such that the cells formed a monolayer the following day. The virus was used starting at a dilution of 1:200 and was subsequently double diluted. Cells were incubated with the viral particles for 2 h at 37°C for virus absorption in serum-free medium. The medium was then removed and a final volume of 200 μL containing equal parts 2% carboxymethyl cellulose (CMC) and FBS-containing medium was used to overlay. Cells were further incubated for 72 h for the plaques to develop. Five percent formaldehyde was used as a fixative for 1 h and 0.25% crystal violet (in 30% methanol) was used for staining cells. The plates were washed three times with 1× PBS, and finally, the plaques obtained were calculated as follows to determine viral titer: PFU/mL = no. of plagues/dilution factor × volume of diluted virus per well.

### Mosquito maintenance, blood feeding, and nanoinjections.

A. aegypti mosquitoes were maintained as described previously (82). Blood feeding was performed by initially starving female mosquitoes for 4 h. Uninfected rabbit blood or blood spiked with CHIKV (10^6^ PFU/mL) was then fed to mosquitoes for a period of 1 h using membrane feeders. Fully fed mosquitoes were distinguished by presence of engorged midgut and separated.

dsRNA injections were performed by injecting 800 ng of total dsRNA into female mosquitoes. For experiments with subsequent blood feeding, injected mosquitoes were fed with CHIKV-infected blood 24 h after nanoinjection. Fed mosquitoes were separated from unfed mosquitoes. A total of 5 individual mosquitoes were then collected in TRIzol and analyzed for cullin-3 and CHIKV genomic RNA levels in qRT-PCR using primers against E1 gene.

### qRT-PCR.

A total of 2 × 10^6^ cells were collected in TRIzol reagent (Invitrogen) for total RNA isolation. Gene expression levels were determined using specific primers with qRT-PCR using QuantiTect SYBR green PCR kit (Qiagen) following manufacturer’s instructions.

For mosquito experiments, 4- to 5-day-old female A. aegypti mosquitoes were fed with either CHIKV-spiked blood (10^6^ PFU/mL) or uninfected blood. Fully engorged mosquitoes were separated. A pool of 5 mosquitoes were then collected in TRIzol every 24 h for 5 days. RNA was extracted from the pool of mosquitoes subjected to qRT-PCR using QuantiTect SYBR green PCR kit (Qiagen) with AeCullin-3-specific primers. Rps17 was used as endogenous control. The experiment was performed three times with three technical replicates. Data were analyzed by 2^−Δ^*^CT^* method and 2^−ΔΔ^*^CT^* and expressed as mean ± standard deviation (SD).

Primer details for qRT-PCR have been listed in Data set S2.

### Statistical analysis.

Statistical analysis was performed using GraphPad Prism 6. All experiments were performed a minimum of three times with three technical replicates. Data are expressed as mean ± SD. The statistical significance of primary screening, MG132 treatment assays, and expression profile for cullin in infected Aedes aegypti mosquitoes was done using one-way analysis of variance (ANOVA) with Dunnett’s multiple-comparison test. Statistical significance of expression profile for viral genomic RNA levels in AeCullin-3-silenced mosquitoes was performed using unpaired Student’s *t* tests. *P* values of <0.05 were considered significant and are represented with an asterisk in the figures.

### Data availability.

Transcriptomics data sets from the current study have been submitted to National Center for Biotechnology Information accession number GSE195852.
